# The effects of different repair methods for a posterior root tear of the lateral meniscus on the biomechanics of the knee: a finite element analysis

**DOI:** 10.1186/s13018-021-02435-0

**Published:** 2021-05-05

**Authors:** Jian-Yu Wang, Yan-Song Qi, Hu-Ri-Cha Bao, Yong-Sheng Xu, Bao-Gang Wei, Yong-Xiang Wang, Bing-Xian Ma, Hui-Wen Zhou, Fei Lv

**Affiliations:** 1 Department of Orthopedics, Inner Mongolia People’s Hospital, No.20 Zhao Wu Da Street, Hohhot, 010017 Inner Mongolia Autonomous Region China; 2 Graduate School of Inner Mongolia Medical University, Hohhot, Inner Mongolia Autonomous Region China

**Keywords:** Finite element analysis, Knee joint, Posterior root tear of lateral meniscus, Biomechanics

## Abstract

**Purpose:**

To explore the impact of different repair methods for a lateral meniscus posterior root tear on the biomechanics of the knee joint using finite element analysis.

**Methods:**

Finite element models of a healthy knee were established on the basis of MRI data from a volunteer using Mimics software, and the validity of the models was tested. The changes in the contact mechanics and kinematics of these finite element models under different repair approaches were then analyzed and compared.

**Results:**

The normal meniscus had the maximum joint contact area, the minimum contact pressure, and the minimum contact stress. When total meniscectomy of the lateral meniscus was performed, the lateral compartment had the minimum joint contact area, the maximum contact pressure and the maximum contact stress. When complete avulsions of the posterior root of the lateral meniscus occurred, the maximum values of contact pressure and contact stress were between those of an intact meniscus and those of a meniscus treated with total meniscectomy. Lateral meniscal root attachment reconstruction by the single-stitch and double-stitch techniques resulted in a significant decrease in joint contact pressure and contact stress, leading to values comparable to those of a normal knee joint, and the double-stitch technique performed better than the single-stitch technique.

**Conclusions:**

Repair surgery for lateral meniscal posterior root avulsions can effectively restore the contact mechanics and kinematics of the knee joint, and the double-stitch technique can result in better clinical outcomes than the single-stitch technique.

## Introduction

Menisci are critical components of the human knee joint. Menisci are crescent-shaped, and the cross-sections of menisci appear wedge-shaped. The meniscus itself is a complex biomechanical component that plays a fundamental role in many joint functions, such as load transfer, shock absorption, proprioception, stability, and lubrication. The meniscal root (MR) refers to the attachment site of the anterior or posterior horn of the meniscus to the intercondylar region of the tibial plateau, and this attachment is essential for maintaining the normal alignment and physiological function of the meniscus. Additionally, meniscal root tears are defined as bony or soft tissue root avulsion injuries or radial tears around the root. The meniscus bears loads on both sides of the tibia and femur during load-bearing tasks, which produce a sliding force that moves the meniscus peripherally. Therefore, the circumferentially arranged collagen fiber bundles inside the meniscus are pulled radially, leading to circular tension that results in “hoop strain”. The meniscal hoop tension can act against the peripherally directed sliding force and transfer the loads to the tibia through the strong connection between the anterior and posterior meniscal roots. The presence of hoop tension allows the axial tibiofemoral loads to uniformly pass through the articular surface and protect the articular cartilage. Meniscal root tears and avulsion injuries lead to the loss of function of the hoop tension, and the loss of protection for the articular cartilage could result in degenerative changes in the knee joint. Posterior root tears of the meniscus (PRTMs) that occur in young people are usually caused by trauma, and PRTMs are usually associated with degenerative joint changes in elderly people. Currently, the impact of a posterior root tear of the lateral meniscus on the biomechanical function and contact mechanics of the knee joint is unclear. Historically, total meniscectomy was a common procedure performed for meniscus tears. With MRI and arthroscopy being widely used in clinical practice and additional biomechanical studies on the meniscus being conducted, considerable advancements in the diagnosis and treatment of meniscus tears have occurred. Currently, there are three main methods for surgically managing meniscus root tears: meniscectomy, meniscal repair, and reconstruction of the posterior root of the meniscus attachment point through the tibial tunnel. It is essential to have a good understanding of the normal biomechanics of the knee to effectively prevent and treat knee joint injuries, and the finite-element method has been proven to be an efficient and accurate way of gaining a deeper understanding of the mechanical properties of living tissue. In this paper, we explore the impact of different repair methods for posterior root tears of the lateral meniscus on the biomechanics of the knee joint using finite element analysis and provide a theoretical basis for the diagnosis and treatment of lateral meniscal posterior root injuries.

## Materials and methods

**Fig. 1:**
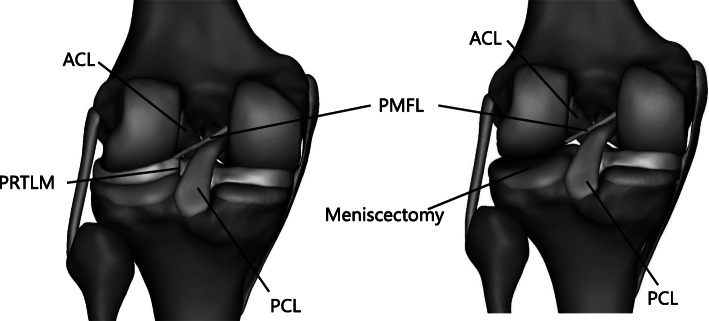
Posterior root tear of the lateral meniscus and lateral total meniscectomy

**Fig. 2:**
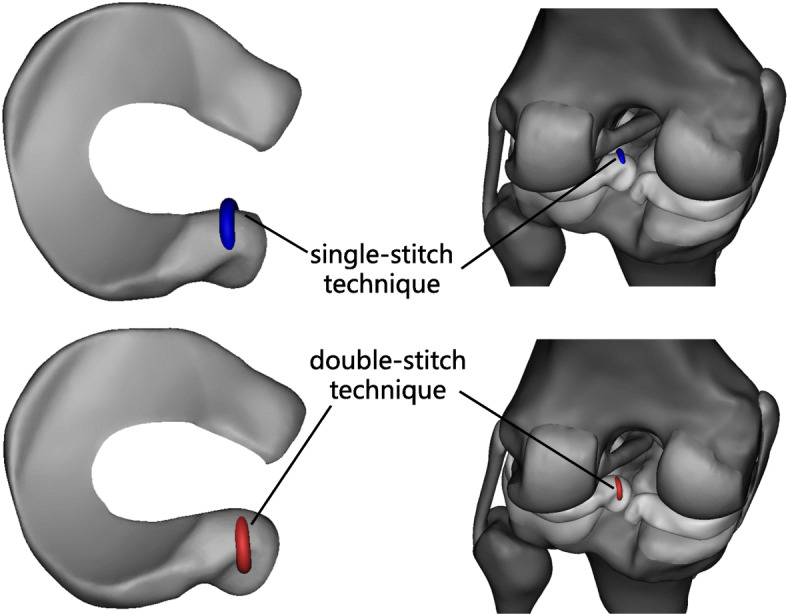
PRTLM attachment point reconstruction with the single-stitch technique and double-stitch technique

The FE model (Figs. 1 and 2) previously created and validated by Bao et al. [1] was adapted in this study to simulate different repair methods for a posterior root tear of the lateral meniscus. The numerical data of the three-dimensional finite element models of the knee were based on MR images of the left knee of a healthy adult volunteer (female, 35 years old). The knee was immobilized in an unloaded and fully extended position. A 3.0-T MR scanner was used to obtain MR images of the knee. The sagittal-plane MR images of the knee were segmented manually, and the three-dimensional surfaces of the model were created using MIMICS (Materialise, Leuven, Belgium). The knee model mainly consisted of the femur, tibia, fibula, articular cartilage, menisci, and the main ligaments of the knee, including the anterior cruciate ligament (ACL), posterior cruciate ligament (PCL), medial collateral ligament (MCL), lateral collateral ligament (LCL), and posterior meniscal femoral ligament (PMFL). The soft tissues and ligaments were meshed with 10-node tetrahedral elements using the finite element meshing software HyperMesh (Altair Inc., Troy, MI). The bones were rigidly constrained and modeled using 2D elements for computational efficiency. This study was approved by the Institutional Review Board, and informed consent was obtained from the candidate.

The material properties of the model were determined from previously published data. Because the stiffness of bone is higher than that of other tissues, the bone was assumed to be rigid. The articular cartilages were defined as isotropic, linear elastic materials with a modulus of 15 MPa and Poisson’s ratio of 0.46 [2]. The menisci were modeled as a transversely isotropic, linearly elastic, homogeneous material with a Young’s modulus of 120 MPa in the circumferential direction and 20 MPa in the axial and radial directions. Poisson’s ratio was 0.2 in both the circumferential and radial directions, and it was 0.3 in the axial direction [3–5]. The roots of the meniscus were defined as 10 mm of meniscal tissue angling down toward the tibial plateau attachment in the intercondylar notch [6]. The meniscal roots were modeled as an isotropic, linear elastic material with a Young’s modulus of 120 MPa and Poisson’s ratio of 0.45 [3, 4, 7]. In accordance with studies performed by other scholars [1, 8], the ligaments were assumed to be nonlinear, hyperelastic, and transversely isotropic fiber materials. No. 5 Fiber Wire was used for the reconstruction of the meniscus root attachment point, with a Young’s modulus of 380,000 MPa and Poisson’s ratio of 0.39 [9].

Six contact pairs were established in the finite element model of the knee joint: the femoral cartilage and the meniscus, the meniscus and the tibial cartilage, and the femoral and tibial cartilage on both the medial and lateral sides. The articulations at the cartilage-cartilage and cartilage-meniscus regions were represented by a finite, sliding, frictionless, hard contact algorithm with no penetration. The tibia and fibula were restricted to six degrees of freedom. The femur was constrained to movement only in the flexion or extension directions to simulate full extension of the knee joint. To compare the obtained results with previously published data, a compressive axial load of 1000 N and a forward thrust load of 134 N were applied to the three-dimensional finite element model of a normal knee joint, and the contact area, contact pressure, contact stress, meniscus displacement, and tibia forward displacement values were calculated for each model.

The accuracy of a finite element analysis of the knee depends on the anatomical truth of the model and the appropriate mathematical definition of each tissue structure in the model [8]. According to a previous study [1], the results obtained from the intact knee joint model were found to be consistent with previously published data by other scholars [10–14]. Thus, the finite element model was considered valid, reasonable, effective, and reliable, so the subsequent steps of this research were performed.

Based on the finite element model of the normal knee joint, a model of a posterior root tear of the lateral meniscus and different repair method models were established. The repair methods included total meniscectomy of the lateral meniscus, for which 3-matic software was used to reconstruct the posterior root of the lateral meniscus attachment point with the single-stitch technique and double-stitch technique through the tibial tunnel (Figs. 1 and 2). A compressive axial load and forward thrust load were applied to the models, and the contact area, contact pressure, contact stress, meniscus displacement, and tibia forward displacement values were calculated for each model. During the study, the PMFL was considered intact.

## Results

### Contact mechanics of various models under axial compressive load

#### Contact pressure and contact stress

**Fig. 3:**
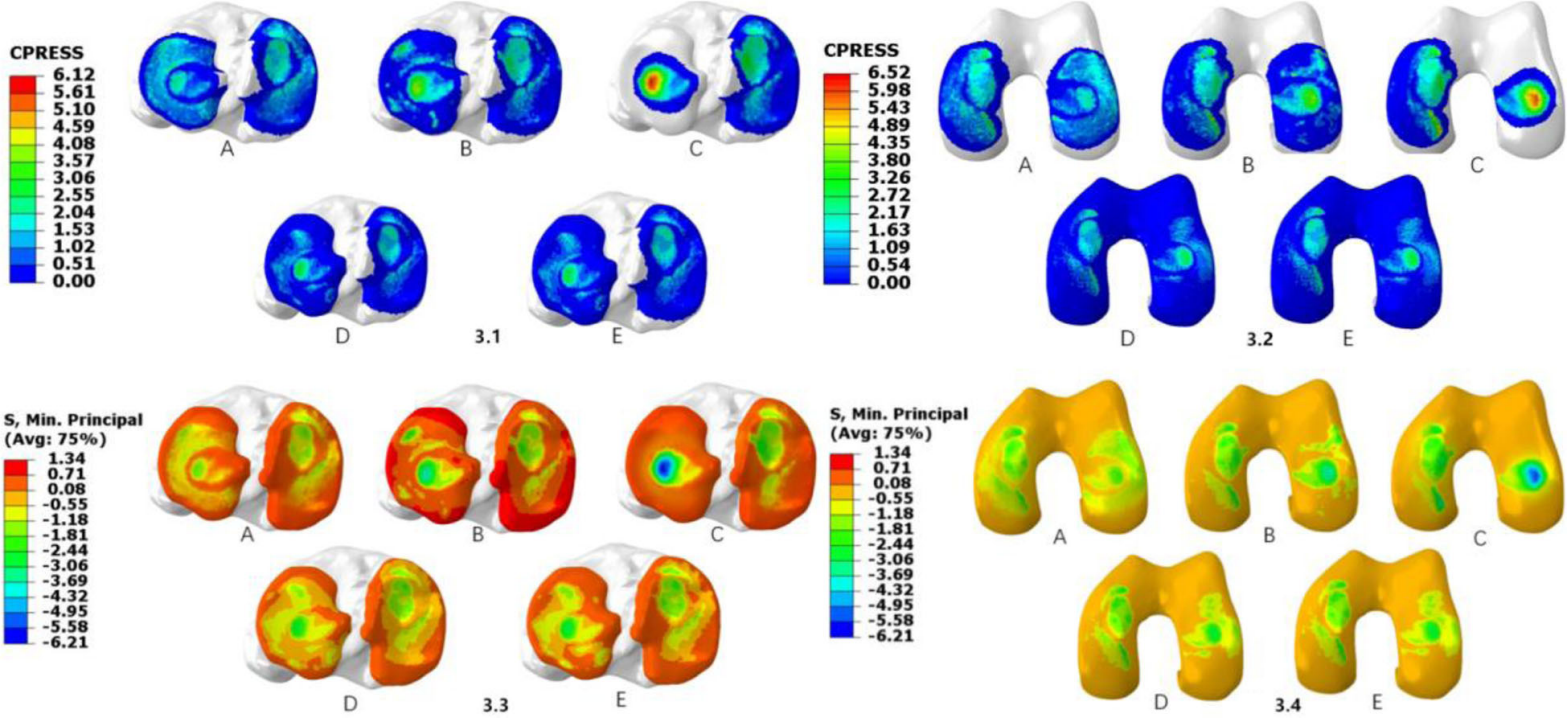
**3.1**–**3.2** Contact pressure distribution in the medial and lateral tibial and femoral articular cartilage under a 1000 N axial compressive load. (A) Intact knee, (B) PRTLM, (C) lateral total meniscectomy, (D) attachment point reconstruction with the single-stitch technique, and (E) attachment point reconstruction with the double-stitch technique. **3.3**–**3.4** Contact stress distribution in the medial and lateral tibial and femoral articular cartilage under a 1000 N axial compressive load. (A) Intact knee, (B) PRTLM, (C) lateral total meniscectomy, (D) attachment point reconstruction with the single-stitch technique, and (E) attachment point reconstruction with the double-stitch technique

Under a 1000 N compression load, the peak contact pressure of the medial compartment of the tibial articular cartilage in the normal knee joint was 2.97 MPa, the peak contact pressure of the lateral compartment was 3.09 MPa, and the maximum stress values in the medial and lateral compartments were 2.36 MPa and 2.25 MPa, respectively. As shown in Table 1, the stress distribution of the medial and lateral compartments of the articular cartilage and meniscus changed significantly with the PRTLM. (1) With the PRTLM, the maximum contact pressures of the medial and lateral tibial cartilage were 3.28 MPa and 3.92 MPa, respectively. The maximum stresses of the medial and lateral tibial cartilage were 2.62 MPa and 3.79 MPa, respectively. (2) When the lateral meniscus underwent total meniscectomy, the maximum contact pressures of the medial and lateral tibial cartilage were 3.35 MPa and 6.12 MPa, respectively. The maximum stresses of the medial and lateral tibial cartilage were 3.37 MPa and 5.54 MPa, respectively. (3) When the posterior root of the lateral meniscus was reconstructed by the single-stitch technique, the maximum contact pressures of the medial and lateral tibial cartilage were 2.762 MPa and 3.75 MPa, respectively. The maximum stresses of the medial and lateral aspects of the tibia were 3.759 MPa and 3.644 MPa, respectively. (4) When the posterior root of the lateral meniscus was reconstructed by the double-stitch technique, the maximum contact pressures of the medial and lateral tibial cartilage were 2.76 MPa and 3.255 MPa, respectively, and the maximum stresses were 3.755 MPa and 3.587 MPa, respectively (Fig. 3).

**Table 1:** Maximum contact pressure and compressive stress of the tibial and femoral articular cartilage (MPa)

Contact pressure/stress	Normal	PRTLM	Complete removed	Single-stitch technique	Double-stitch technique
Medial tibial cartilage peak contact pressure	2.97	3.28	3.35	2.762	2.76
Maximum contact stress	2.36	2.62	3.37	3.759	3.755
Lateral tibial cartilage peak contact pressure	3.09	3.92	6.12	3.75	3.255
Maximum contact stress	2.25	3.79	5.54	3.644	3.587
Femur cartilage peak contact pressure	3.84	4.56	6.52	4.925	4.903
Maximum contact stress	3.11	4.43	6.21	4.909	4.895

#### Contact area

**Fig. 4:**
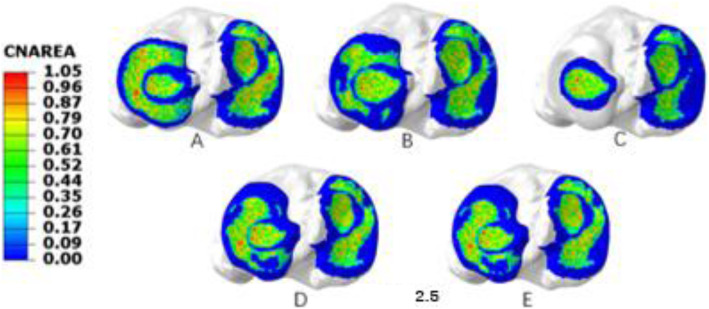
Contact area distribution in the medial and lateral tibial articular cartilage under a 1000 N axial compressive load. (A) Intact knee, (B) PRTLM, (C) lateral total meniscectomy, (D) attachment point reconstruction with the single-stitch technique, and (E) attachment point reconstruction with the double-stitch technique

The total contact area of the tibial articular cartilage of the normal knee was 1044.54 mm^2^. The total contact area of the lateral compartment of the knee joint decreased from 512.286 mm^2^ with the complete meniscus to 487.992 mm^2^ with the PRTLM. The total contact area of the medial compartment decreased from 532.254 to 512.297 mm^2^. The contact area of the lateral compartment was 244.914 mm^2^ when the lateral meniscus was completely resected, while the medial contact area was 423.486 mm^2^. The contact area of the lateral compartment was restored to 493.679 mm^2^, and the contact area of the medial compartment was 537.324 mm^2^ when the single-stitch technique was performed at the attachment point of the posterior root. The contact area of the cartilage returned to mostly normal with the double-stitch technique at the attachment point, and the contact areas of the medial and lateral compartments were 568.007 mm^2^ and 508.678 mm^2^, respectively (Fig. 4).

#### Radial displacement of the meniscus

Under a 1000 N axial compression load, the following changes occurred. (1) Radial displacement of the lateral meniscus of the normal knee joint was not obvious. (2) With the PRTLM, radial displacement of the body and the posterior horn was observable, as the maximum displacement was 5.44 mm outward and 7.58 mm backward, and displacement of the front horn was not obvious. (3) With the single-stitch technique at the posterior root of the lateral meniscus attachment point, the maximum displacement was 2.53 mm outward and 5.14 mm in the backward direction. (4) The maximum displacement was 1.244 mm in the outward direction and 2.817 mm in the backward direction with the double-stitch technique at the posterior root of the lateral meniscus attachment point. The magnitude of displacement of the medial meniscus did not obviously change in the aforementioned conditions.

#### The influence of a forward thrust on each model of the knee joint

Under the forward thrust of 134 N, there were no significant differences in the distance of forward movement of the tibia among the models, all of which were 5.09 mm.

## Discussion

A root tear of the meniscus was found in both the anterior and posterior attachment points of the meniscus, but a posterior root tear was more common. The medial meniscus is more likely to be torn because its range of motion is relatively small, and large stresses are placed on the medial meniscus when the knee is loaded. For patients with an ACL injury, the incidence of PRTLM is higher than that of PRTMM [15]. The lateral meniscus has more mobility and remains stable when securely attached to the root to the tibial plateau. Damage to the posterior root of the lateral meniscus results in a decline in the integrity of the circular collagen fibers and the transformation of the ring stress in the meniscus [16]. As a result, tibiofemoral joint stress obviously increases [17, 18].

PRTLMs are associated with ACL injuries [6]. Arthur et al. [19] assessed 559 knee joints, and 8% of the patients with a PRTLM had an ACL injury. However, in the cases with an intact ACL, only 0.8% of the patients had a PRTLM. James et al. [20] reported 772 cases of meniscal tears associated with an ACL rupture, more than 50% of which were located in the posterior root of the lateral meniscus. Previous studies have shown that in cases treated with ACL reconstruction, the proportion of cases with a PRTLM ranges from 7 to 12% [21, 22]. An imaging study by Jeffrey et al. [15] showed that in cases of an ACL injury, the incidence of a PRTLM was significantly higher than that of a medial injury. In another study of ACL injuries, 10% of the patients had a PRTLM, and only 3% of the patients had a posterior root tear of the medial meniscus.

At present, the treatments for PRTLM include conservative and surgical treatments, but there is no treatment standard. For a PRTLM, simple reconstruction of the ACL, partial excision, or conservative treatments are suggested. Conservative treatments include the injection of drugs into the articular cavity to protect the articular cartilage and the use of NSAIDs or physiotherapy to protect the meniscus and meniscus root tears, thus delaying the development of meniscus tears. Shelbourne et al. [23] followed up with patients with a PRTLM conservatively for an average of 10 years; the results showed that there were no significant differences in the subjective or objective measures of the knee joint except for the narrowing of the lateral joint space in these patients compared with a control group. The authors also suggested that the biomechanical changes in the knee joint that occur with a PRTLM were similar to those that occur with a posterior root tear of the medial meniscus, so the authors suggested that the posterior root tear of the lateral meniscus is repaired at the same time the ACL is reconstructed. Robert et al. [24] showed that the biomechanical properties of the meniscus could be recovered by repairing the PRTLM. Jin et al. [25] performed ACL reconstructions and all-inside sutures for meniscal root tears in 25 patients. Eighteen months following the surgery, all patients exhibited favorable clinical results. For both conservative and surgical treatments, the purpose is to restore the stability of the root of the lateral meniscus and to delay the degeneration of the articular cartilage. Therefore, it is very important to study PRTMs in the clinic.

In this study, a previously constructed finite element model of the knee developed with data from a healthy, young female was used. The contact pressure and contact area of the normal knee model were compared with previously published experimental data [10–14]; we found that the results of the model are comparable to previously published data, confirming that the model can be used reasonably. With a PRTLM, the destruction of the annular collagen fiber bundle and the axial displacement prevent axial loads from passing relatively uniformly through the joint surface, so the joint surface stress is concentrated, and the maximum contact pressure and stress are significantly large. The results of this study showed that with a PRTLM, the contact area of the meniscus with femoral and tibial articular cartilage decreased, the direct contact area between the articular cartilage increased, and the total joint contact area decreased (1000.289 mm^2^), resulting in a significant increase in the contact pressure and stress of the articular cartilage to 26.9% and 68.4%, respectively. When total meniscectomy of the lateral meniscus was performed, the pressure and stress changes in the medial and lateral tibial articular cartilage were the largest. It has been reported that when total meniscectomy of the lateral meniscus is performed, the contact area of the lateral compartment of the knee joint decreases by approximately 50% compared with that of a normal knee joint, and the contact pressure peak increases by approximately 100–300% [11, 26, 27], which is consistent with the results of this study. At this time, the maximum contact pressure and stress of the lateral cartilage of the tibia increased by 98.1% and 146.2%, respectively, compared with those of the normal knee joint. When the posterior root of the lateral meniscus was reconstructed by the single-stitch technique and double-stitch technique, the maximum contact pressure in the lateral compartment increased by 21.6% and 62.0%, respectively, and the maximum stress increased by 5.3% and 59.4%, respectively, compared with those of the normal knee joint; however, the maximum contact area was comparable to that of the intact state and only decreased by 3.6% and 0.7%, respectively. In the normal meniscus, the joint contact area was the largest, and the contact pressure and contact stress were the smallest. When total meniscectomy of the lateral meniscus was performed, the contact area of the lateral compartment was the smallest, and the contact pressure and stress were the largest. With a PRTLM, the maximum contact pressure and stress were between those of the intact knee and those of the knee treated with total meniscectomy of the lateral meniscus, and the lateral compartment pressure and stress remained relatively small. Posterior root attachment point reconstruction with the single-stitch technique and double-stitch technique can effectively reduce the joint contact pressure and stress and restore the joint contact area to levels similar to those of the intact meniscus. The double-stitch technique results in better clinical outcomes than does the single-stitch technique, with outcomes nearly identical to those of a fully intact meniscus. Clinically, the extent of degenerative changes in the knee joint after total lateral meniscectomy is severe [26]. However, patients with PRTLM do not have significant osteoarthritis. This finding suggests that the residual lateral meniscus still has a conductive load function. With a PRTLM, the radial displacement of the body and the posterior horn of the lateral meniscus increases under axial load, which means that the joint space is narrowed, and the mechanical environment of the lateral compartment is changed. The displacement of the tibia under the forward thrust had no obvious relationship with the state of the meniscus, and the magnitude of displacement was nearly the same.

## Conclusions

The PRTLM can be repaired by surgery, and the contact mechanics and kinematics of the knee joint can be effectively restored to levels similar to those of an intact knee joint. Compared with the single-stitch technique, the double-stitch technique is more effective and plays an important role in restoring meniscal stability, protecting articular cartilage and delaying joint degeneration.

## Data Availability

Please contact author for data requests.

## Abbreviations

MR: Meniscal root
PRTM: Posterior root tears of the meniscus
PRTLM: Posterior root tear of lateral meniscus
ACL: Anterior cruciate ligament
PCL: Posterior cruciate ligament
MCL: Medial collateral ligament
LCL: Lateral collateral ligament
PMFL: Posterior meniscal femoral ligament
